# Accessory signals in protein translocation

**DOI:** 10.18632/aging.101435

**Published:** 2018-04-28

**Authors:** Katja G. Hansen, Felix Boos, Johannes M. Herrmann

**Affiliations:** 1 Cell Biology, University of Kaiserslautern, 67663 Kaiserslautern, Germany

**Keywords:** chaperones, mitochondria, protein folding, protein translocation, signal sequences

A large fraction of the polypeptides synthesized in the cytosol of eukaryotic cells carry targeting signals to direct them to specific cellular compartments [1]. Proteins that reach their target compartment in an unfolded conformation typically display their targeting signals at their N-termini. Examples are the signal sequences of secretory proteins, the matrix targeting sequences (MTSs, also called presequences) of mitochondrial proteins, the transit peptides of chloroplast proteins as well as bacterial leader peptides. These N-terminal targeting signals are necessary and sufficient for protein translocation. If fused to almost any protein, they reliably drag their fusion partner into their respective compartment. In most cases, they are removed from the mature segments of the proteins by processing peptidases subsequent to the translocation reaction.

**Figure 1:**
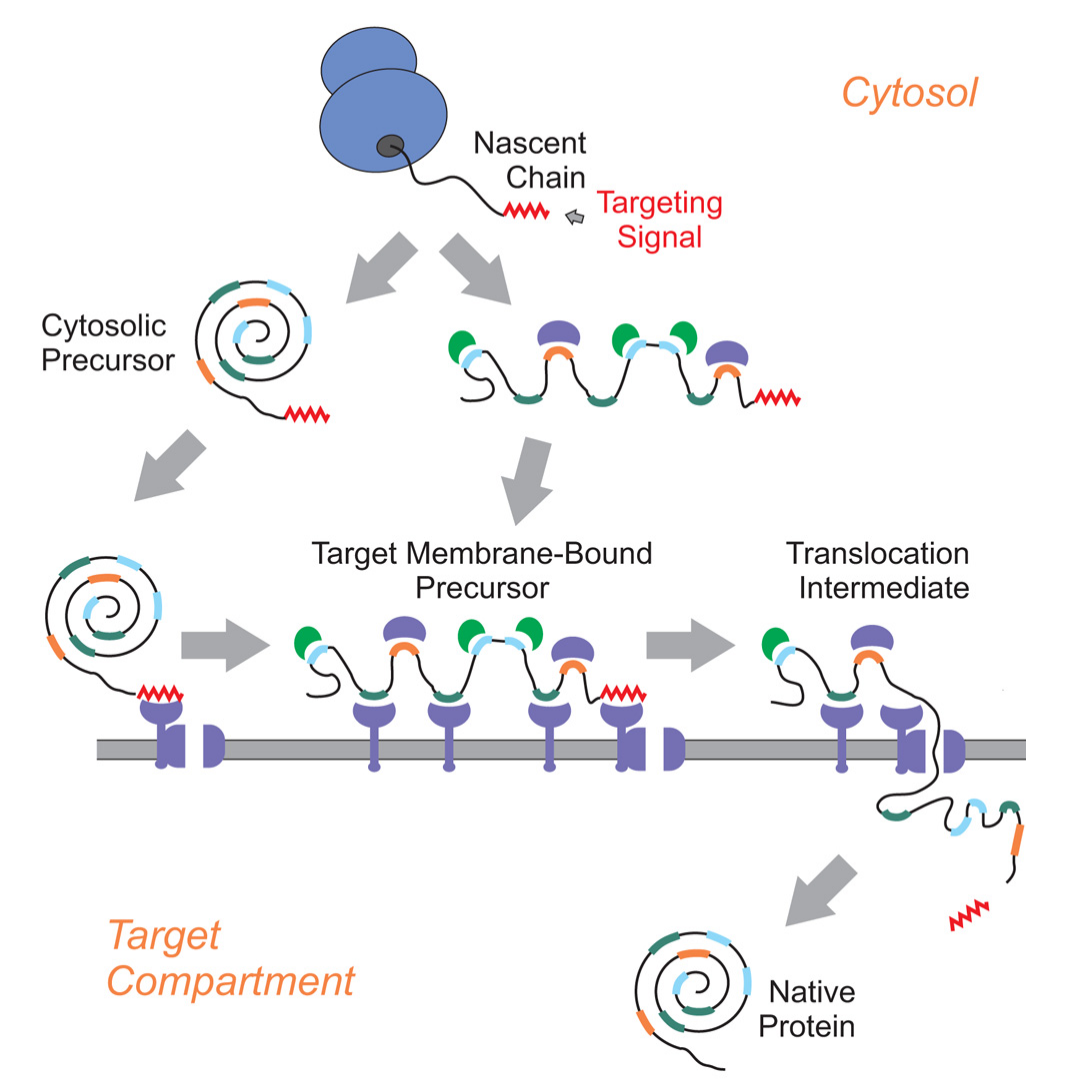
**Accessory signals in protein translocation.**

A number of recent studies, however, showed that the information relevant for protein targeting to the mitochondrial matrix is not only restricted to the N-terminal targeting sequences, but additional accessory signals in the mature regions of precursor proteins can considerably influence protein translocation [2,3]. While these signals are dispensable for targeting per se, they appear to be decisive for the efficiency and velocity of the translocation reaction. These internal sequences presumably fulfill different functions, in particular in the association with cytosolic chaperones and with receptor proteins exposed on membrane surfaces. These accessory signals thereby counteract the folding or even drive the untangling of (loosely) folded cytosolic precursors (Fig. 1).

The MTSs of mitochondrial proteins form amphiphatic helices with one hydrophobic and one positively charged surface. Recently, it was observed that segments of similar characteristics are scattered along the mature sequences of many mitochondrial proteins [2]. Given their similarity to presequences, they were named internal matrix targeting signal-like sequences (iMTS-Ls). Prediction programs such as TargetP, normally designed to identify MTSs, can easily be utilized to recognize iMTS-Ls [2]. The iMTS-Ls bind to the mitochondrial surface receptor Tom70. Tom70 belongs to the tricopeptide repeat (TPR) protein family, members of which serve as cofactors of Hsp70 and Hsp90 chaperones. Thus, mitochondrial precursor proteins obviously contain defined binding sites for a mitochondrial surface protein and for chaperones spread along their sequence which, at least *in vitro*, were found to be critical for the import efficiency of these proteins [2]. In their function as Tom70 recognition motifs, iMTS-Ls are similar to the internal targeting sequences that are known from mitochondrial carrier proteins, a family of inner membrane transporters that lacks N-terminal MTSs. The relevance of iMTS-Ls for mitochondrial protein import explains previous observations based on chimeric fusion proteins in which presequences and mature domains were exchanged: these studies revealed a large influence of the mature region on the import efficiency and on their Tom70-dependence [3]. The presence of iMTS-Ls in most matrix proteins suggests that these sequences play a very general role in early steps of mitochondrial protein targeting.

Interestingly, elegant studies, which analyzed the folding behavior of bacterial secretory proteins revealed that the mature regions of these proteins generally fold with very slow kinetics, presumably in order to ease their membrane translocation [4]. Slow folding of secretory proteins is the result of specific properties of their mature regions, which often contain a large proportion of disordered regions. These properties are so prevalent that they can be used to identify secretory proteins just on the basis of their mature sequence, as convincingly demonstrated by algorithms such as MatureP [5]. In addition, the binding of chaperones and folding factors can further increase the translocation competence of bacterial proteins. Similar features presumably apply to eukaryotic secretory proteins, at least to those that are translocated posttranslationally [6,7].

Whether the features in regions that slow down folding in secretory proteins are similar to those of iMTS-Ls in mitochondrial proteins is not known. However, the distribution of iMTS-Ls is not restricted to mitochondrial proteins. Also many secretory precursors (but also cytosolic proteins) contain sequences of the same properties. Whether these sequences also serve as ‘stepping stones’ for surface receptor proteins and cochaperones is not known. However, TPR proteins of similar structure as Tom70 are also present at the surfaces of the ER and of peroxisomes (e.g. Sec72 and Pex5) as well as in the cytosol (e.g. Sti1), and might indeed serve a Tom70-like function.

Helices with opposing hydrophobic and positively charged surfaces, as characteristic for iMTS-Ls, are known to absorb to (negatively charged) membrane surfaces. Thus, it is conceivable that their direct association with low affinity to lipid bilayers contributes to the slow folding behaviors of precursor proteins. Such a lipid-mediated binding would hardly be selective for one specific target membrane, making so far undiscovered mechanisms necessary that allow the transfer of precursors between different cellular membrane surfaces. Obviously, even more than 40 years after the discovery of signal sequences on secretory proteins, central aspects of the cellular protein targeting processes are still to be discovered.
